# Implementing a Transition Program from Paediatric to Adult Services in Phenylketonuria: Results after Two Years of Follow-Up with an Adult Team

**DOI:** 10.3390/nu13030799

**Published:** 2021-02-28

**Authors:** Maria Peres, Manuela F. Almeida, Élia J. Pinto, Carla Carmona, Sara Rocha, Arlindo Guimas, Rosa Ribeiro, Esmeralda Martins, Anabela Bandeira, Anita MacDonald, Júlio C. Rocha

**Affiliations:** 1 Centro de Genética Médica, Centro Hospitalar Universitário do Porto (CHUP), 4099-028 Porto, Portugal; maria_cpperes@gmail.com (M.P.); manuela.almeida@chporto.min-saude.pt (M.F.A.); eliajoana17@gmail.com (É.J.P.); carla.carmona@chporto.min-saude.pt (C.C.); 2 Faculdade de Ciências da Nutrição e Alimentação, UP, 4150-177 Porto, Portugal; 3 Centro de Referência na área de Doenças Hereditárias do Metabolismo, Centro Hospitalar Universitário do Porto-CHUP, 4099-001 Porto, Portugal; saraisabelrocha@gmail.com (S.R.); arlguimas@gmail.com (A.G.); rocrff@gmail.com (R.R.); esmeralda.g.martins@gmail.com (E.M.); anabela.ol.bandeira@sapo.pt (A.B.); 4 Unit for Multidisciplinary Research in Biomedicine, Abel Salazar Institute of Biomedical Sciences, University of Porto-UMIB/ICBAS/UP, 4050-313 Porto, Portugal; 5 Birmingham Women’s and Children’s Hospital, Birmingham B4 6NH, UK; anita.macdonald@nhs.net; 6 Centre for Health Technology and Services Research (CINTESIS), 4200-450 Porto, Portugal; 7 Nutrition & Metabolism, NOVA Medical School, Faculdade de Ciências Médicas, Universidade Nova de Lisboa, 1169-056 Lisbon, Portugal

**Keywords:** phenylketonuria, transition to adult care, metabolic control, adherence

## Abstract

We aimed to report the implementation of a phenylketonuria (PKU) transition program and study the effects of follow-up with an adult team on metabolic control, adherence, and loss of follow-up. Fifty-five PKU patients were analysed in the study periods (SP): 2 years before (SP1) and after the beginning of adult care (SP2). Retrospective data on metabolic control and number of clinic appointments were collected for each SP, and protein intakes were analysed. In SP2, three patients (6%) were lost to follow-up. There was a small but statistically significant increase in median number of annual blood spots from SP1 to SP2: 11 (7–15) vs. 14 (7–20); *p* = 0.002. Mean ± SD of median blood Phe remained stable (525 ± 248 µmol/L vs. 552 ± 225 µmol/L; *p* = 0.100); median % of blood Phe < 480 µmol/L decreased (51 (4–96)% vs. 37 (5–85)%; *p* = 0.041) and median number of clinic appointments increased from SP1 to SP2: (5 (4–6) vs. 11 (8–13); *p* < 0.001). No significant differences were found regarding any parameter of protein intake. Our results suggest that the implementation of an adult service was successful as impact on metabolic control was limited and attendance remained high. Continuous dietetic care likely contributed to these results by keeping patients in follow-up and committed to treatment.

## 1. Introduction

Phenylketonuria (PKU) is an inborn error of phenylalanine (Phe) metabolism [1]. Early diagnosis and implementation of a Phe restricted diet have drastically improved clinical prognosis [1], and the PKU European guidelines state that treatment and follow-up should be maintained in adulthood [2]. However, the growing adult population with PKU is still understudied and the impact of PKU in the ageing process is unknown [3]. PKU in adulthood has been associated with neurological and neuropsychiatric issues such as deficits in executive function, tremor, depression, anxiety, and white matter abnormalities [4,5,6], but comorbidities affecting other body systems are also reported [7,8,9].

Adherence to the Phe-restricted diet becomes increasingly difficult from adolescence onwards [10]. Obstacles include poor palatability of protein substitutes [11,12], limited food choice, difficulties with food preparation, and severe restrictions on social life [13,14,15]. This can be particularly challenging for patients with classical PKU with a low tolerance of Phe and natural protein, who still need a very strict diet in adulthood [2]. Studies have consistently found deterioration of metabolic control with age in PKU [16,17,18,19]. A study in the USA highlighted that while most patients up to 12 years old had average Phe levels within target range, the same was true for only 49% of teenagers, 41% of young adults and 31% of patients ≥30 years old [20]. A high percentage of adult patients cease contact with their metabolic team, they stop attending clinic appointments, and are consequently lost to follow-up [19,20,21]. Generally, female are more likely than male patients to adhere to some dietary restriction and remain in follow-up, which is probably associated with concerns about maternal PKU syndrome [19].

It is appropriate that adults with PKU should expect to be treated in an adult environment, with adult teams trained to manage their issues and comorbidities [22]. However, many adult clinics do not offer a service to adults with PKU [23]. Thereby patients remain in paediatric care, commonly ‘breaching’ the ‘follow-up’ rules of paediatric hospitals [19] and are left without specialised medical care due to lack of experienced adult physicians. Other patients may have been discharged to their General Practitioner and community services as their paediatric hospitals are no longer able to support them. Fortunately, some new adult services are currently being commissioned but some older patients may go years without receiving appropriate follow-up, and many may abandon their dietary treatment. Patients who discontinue treatment are often unwilling or unable to go back on the diet later in life, while adult teams may struggle to contact them so they can resume specialist care [24]. Failure to follow-up adult patients with PKU will also increase the probability of metabolic control deterioration which will be a lost opportunity to further understand the impact of the ageing process in PKU. 

Careful transition from paediatric to adult metabolic services is imperative, enabling the patient and their family to develop confidence and build a relationship with their adult team. Failure can lead to patient dissatisfaction and poor engagement [25]. The European PKU guidelines [2] recommend starting the transition process at 12 years of age. The transfer to an adult treatment centre commonly occurs between the patient age 16 to 18 years, although the optimal time for transfer should be determined for each case depending on individual circumstances and patient maturity [2]. Because patients with PKU may have decreased autonomy [26], a later transfer may be advantageous as they are likely to be more mature and self-sufficient, making it easier to handle changes in healthcare provision.

The transition process should aim to develop the practical skills and knowledge required by an adolescent, allowing them to become gradually more responsible for managing their own condition while their caregivers slowly step down [15]. Transition should continue even after the transfer between hospitals, as it is better if the first few appointments are conducted jointly by both members of the paediatric and adult metabolic teams. These aspects of transitional care have been associated with better outcomes and could maximize retention in adult services [27]. Some additional useful considerations that will assist in the transition process were recently published as a recommendation from the European PKU Guidelines Group [28].

Little is known about the effect of transition on PKU outcome. It is difficult to study as a separate entity as management guidelines and treatments are constantly evolving with new therapies such as tetrahydrobiopterin (BH_4_) and more recently Pegvaliase [29]. However, absence of blood Phe deterioration, patient satisfaction and patients remaining in follow-up post-transition are usually seen as encouraging, and are useful indicators of transition outcome [30,31]. 

In this paper we aim to report the establishment of paediatric and adult services for PKU care at our centre, and the implementation of a transition program from the paediatric to the adult setting. Specifically, we study the effects of the first two years of follow-up by an adult team on the quality of metabolic control, patient adherence to treatment, and loss of follow-up.

## 2. Materials and Methods

### 2.1. Follow-Up Care Protocol for Patients with PKU

Newborn screening for PKU was implemented in Portugal in 1979. It was located at *Centro de Genética Médica*, where a team of nutritionists, psychologist and geneticist provided treatment for patients with PKU. In addition, a paediatrician from the nearest paediatric hospital (*Hospital Central Especializado de Crianças Maria Pia*) visited *Centro de Genética Médica*, but treatment was mainly managed by nutritionists.

Patients > 18 years were left without specialized adult medical care, as they were ‘too old’ to see a paediatrician according to hospital policy. These patients were kept under the supervision of nutritionists, psychologist and geneticist. Moreover, a group of adult physicians and nurses from *Centro Hospitalar Universitário do Porto* who had received training in inherited metabolic disorders, started working with *Centro de Genética Médica* in 2009. In the following years, some adult patients with PKU were assigned to one physician, but medical follow-up was still infrequent.

In 2013, *Centro de Genética Médica* and the paediatric hospital were merged with *Centro Hospitalar Universitário do Porto* and moved to a new facility in 2016. Structured paediatric and adult services for PKU care were soon created and medical follow-up became more frequent. The paediatric team included metabolic paediatricians and nurse who cared for patients approximately up to 18 years old at the paediatric hospital. In the adult team, the physicians and nurses were able to slowly incorporate more patients in follow-up at the adult hospital. This went on until eventually, every adult patient with PKU had access to specialized medical care that focused on health promotion, prevention and management of co-morbidities. The same nutritionists, psychologist and geneticist who had provided care until then became part of both paediatric and adult teams and have continued to follow-up all the patients ever since the merge.

In the paediatric as well as in the adult care protocol, each clinic visit now consisted of multiple appointments (medical, nutritional, psychological) and the collection of blood spots for Phe measurement. We were, thereby, able to optimize each visit and reduce the frequency of clinic visits, thus minimising burden on patients. Patients also undergo an annual nutritional status evaluation that involves anthropometric assessments, body composition analysis by bioelectrical impedance and measurement of several blood markers of nutritional status.

**Figure 1:**
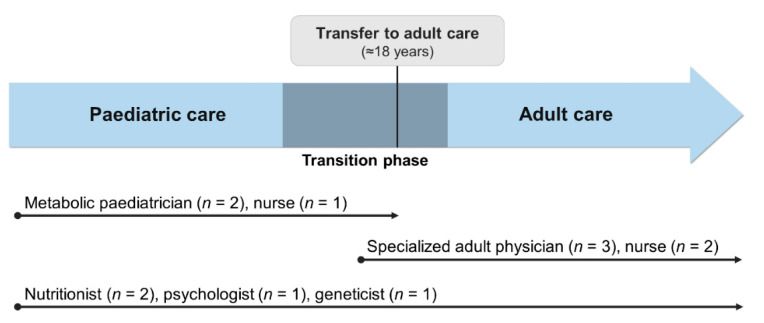
Representation of the current transition process and the multidisciplinary approach throughout all life stages at *Centro Hospitalar Universitário do Porto*. As shown above, only the medical and nursing staff change during transition to adult services. The same nutritionists, psychologist and geneticist are included in both paediatric and adult teams and are responsible for the follow-up of patients at all ages.

Once the paediatric and adult services were fully established, the first patients to experience a transition from their paediatric to their adult physician were transferred in 2015. We have since implemented a more structured transition protocol (Figure 1). Transition is now initiated around 14 years old, as patients are encouraged and educated to become more involved in the management of their condition and undertake self-care. We educate patients and families about the need for transition and provide information about the adult team and follow-up care in the adult service. We also reassure them there will still be no costs to pay for protein substitutes and special low protein foods. Patients meet their new adult physician once before transfer at the paediatric hospital, in a joint consultation with their paediatrician. Transfer occurs at approximately 18 years old, although the exact timing is agreed with each patient and caregivers.

### 2.2. Participants

We identified all patients with PKU at *Centro de Genética Médica/Centro Hospitalar Universitário do Porto* who started follow-up with an adult physician until 2015, and who had received treatment at our centre for a minimum of 2 years prior (*n* = 67). The date of the first medical appointment with the adult physician was used to define the beginning of adult care for each patient.

### 2.3. Study Design

**Figure 2:**
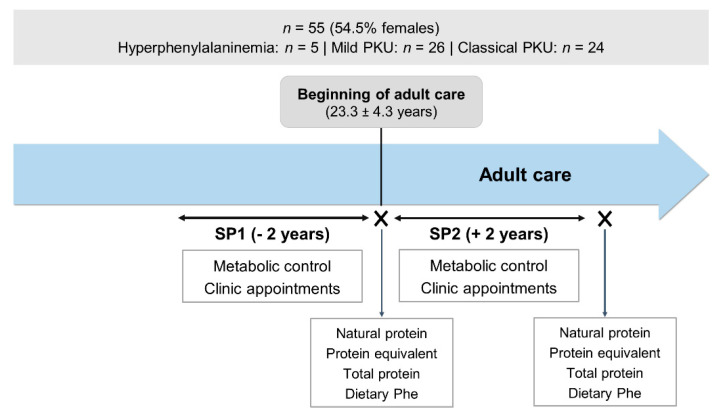
Study design. PKU, phenylketonuria; SP1, study period 1; SP2, study period 2; Phe, phenylalanine.

This was an observational, retrospective, longitudinal study. All data was gathered from patient clinical records. Retrospective data regarding metabolic control, and frequency of clinic appointments were collected and analysed in two different study periods (SP): 2 years before and after the start of adult care (SP1 and SP2, respectively). Data on natural protein, protein equivalent from protein substitute, total protein and dietary Phe intakes from when patients entered adult care and after SP2 were compared. The study design is illustrated in Figure 2.

### 2.4. Data Collection

#### 2.4.1. Metabolic Control

Fasting blood Phe concentrations were measured from dried blood spots and analysed using tandem mass spectrometry. Patients were requested to take at least one blood spot at home every month, according to national consensus [32]. Patients could occasionally be asked to do this more frequently depending on metabolic control (for example, if there were sudden changes or fluctuations in blood Phe), changes in dietary prescriptions, during illness, among other reasons. The annual number of returned blood spots, the median blood (Phe) and percentage of measurements within the treatment range (<480 µmol/L [32]) were calculated for each patient in SP1 and SP2. Patients who had a BH_4_ loading test in SP1 or SP2 were identified, as this may have affected metabolic control.

#### 2.4.2. Frequency of Clinic Appointments

Electronic clinical records were analysed to determine the number of medical appointments (with a paediatrician in SP1, or an adult physician in SP2) and nutrition appointments attended and not attended for each SP. Each medical and nutrition appointment was identified and calculated separately, even though they were usually scheduled for the same day. Patients who did not return to clinic after the first appointment with an adult physician were considered lost to follow-up.

#### 2.4.3. Protein Intake

Protein intake at the start of adult care was estimated at the last nutrition appointment before the first encounter with the new adult physician. Protein intake after SP2 was estimated at the first nutrition appointment after the end of SP2.

Diet history questionnaires and body weight measurements were performed to calculate daily protein intake. Natural protein (g/kg/day), protein equivalent from protein substitute (g/kg/day), total protein (g/kg/day) and Phe (mg/day) intakes were included in data collection. Anthropometric assessments were performed as previously described [33]. However, only weight (which was assessed with a single measurement) was used to quantify protein intakes in g/kg body weight.

#### 2.4.4. Ethical Statement

All the data collected had ethical approval from the Ethics Committee of *Centro Hospitalar Universitário do Porto, EPE*, on 18th May 2015, to the investigation project TNSPKU (Trends in nutritional status of patients with phenylketonuria), with the reference 2015.101 (092-DEFI/087-CES). Patients gave written informed consent.

#### 2.4.5. Statistical Analysis

Statistical analysis was done using IBM SPSS Statistics 25 for Windows. The Kolmogorov-Smirnov test was performed to verify the normal distribution of variables. Categorical variables were presented as absolute values or percentages, while continuous variables were presented as means ± SDs or as medians (P_25_–P_75_). Paired t-test and Wilcoxon test were used to identify differences between variables with normal and non-normal distribution, respectively. Statistical significance was considered when *p* < 0.05.

## 3. Results

### 3.1. Patient Demographics

A total of 12 patients were excluded: 10 were late diagnosed and had inconsistent dietary adherence, whilst 2 initiated sapropterin treatment during the study period, which may have influenced metabolic control.

Therefore, 55 patients (54.5% females) were studied. The disease severity was classified according to the Portuguese consensus [32], using neonatal blood (Phe): 5 patients (9.1%) had hyperphenylalaninemia (blood (Phe) < 360 µmol/L), 26 patients (47.3%) mild PKU (blood (Phe) ≥ 360 µmol/L and ≤1200 µmol/L) and 24 patients (43.6%) classical PKU (blood (Phe) > 1200 µmol/L). Mean ± SD age at the start of adult care was 23.3 ± 4.3 years (range 18–33 years).

Table 1 describes gender, disease severity, age and year of the first appointment with an adult physician, and enrolment to a BH_4_ loading test. Patients initiated follow-up with an adult physician between 2011 and 2015 but more than half started in 2013 (*n* = 31). No BH_4_ loading tests were performed in SP1, but they were performed in almost half of the patients (*n* = 24) in SP2. No patients started pharmacological treatment in this period.

Three patients (6% of the total sample; 2 mild PKU females and 1 classical PKU male) did not return to clinic after the first appointment with the adult physician. They were considered lost to follow-up, resulting in a final sample of 52 patients. There was one pregnancy in SP2, which was unplanned and unfortunately resulted in miscarriage.

### 3.2. Blood Phenylalanine Control

Blood Phe control during the study is presented in Table 2. There was a 27% increase in the median annual number of analysed blood spots from SP1 to SP2 (11 (7–15) vs. 14 (7–20); *p* = 0.002), while the mean ± SD of the median blood (Phe) remained stable (525 ± 248 µmol/L vs. 552 ± 225 µmol/L; *p* = 0.100). The percentage of blood Phe measurements within the treatment range (<480 µmol/L) significantly decreased in SP2 (51 (4–96)% vs. 37 (5–85)%; *p* = 0.041).

After stratifying the sample and analysing only the subjects who remained in follow-up and did not have a BH_4_ loading test in SP2 (*n* = 28), the annual number of blood spots increased by 18%, but it did not reach statistical significance (11 (8–16) vs. 13 (6–19); *p* = 0.374). In contrast, there was a slight but significant increase in the median blood Phe in this subset of patients (375 (236–672) µmol/L vs. 412 (319–728) µmol/L; *p* = 0.001), while the percentage of blood Phe measurements within the therapeutic range decreased, and was statistically significant (91 (16–100)% vs. 77 (12–97)%; *p* = 0.012).

### 3.3. Follow-Up

As demonstrated in Table 3, the frequency of nutrition and medical appointments increased in SP2. Respectively for SP1 vs. SP2, patients had 5 (3–6) vs. 7 (5–8) nutrition appointments (*p* < 0.001) and 0 (0–1) vs. 4 (3–5) medical appointments (*p* < 0.001). In fact, 73% of patients did not have any scheduled appointments with a physician in SP1 (data not shown). A small but significant increase was also seen in the number of total missed appointments, with a median attendance of 100% in SP1 to 91% in SP2 (data not shown), but overall attendance was excellent in both SP1 and SP2.

### 3.4. Dietary Intake

Table 4 shows that protein and Phe intakes at the beginning of adult care and after SP2 were similar. There was a slight decrease in total protein intake and Phe daily intake increased, but these differences did not reach statistical significance.

## 4. Discussion

In this retrospective review of follow-up by an adult team for two years, patients remained in follow-up with a slight deterioration in blood Phe control. Most patients did not have specialized medical care in the two years prior to being assigned an adult physician. However, the same nutritionists provided continuous care both before and after structured paediatric and adult services were in place, and overall adherence was good throughout the study. Very few patients were lost to follow-up and frequency of blood Phe testing increased in the adult setting. 

There were only 6% of patients lost to follow-up after the first appointment with an adult physician. In another study, one adult clinic reported losing contact with 11% of patients with PKU, although this referred to a 10-year period [31]. The number of patients who did not return to our clinic was too small to see any gender differences. We had predicted that more male patients would abandon care compared with females because of maternal PKU concerns, similarly to what was described in an international survey [19]. Structured transition programs in services for other disorders have reported contrasting results. In one paper, all patients with type 1 diabetes remained in follow-up three years after transfer [34]. Other programs documented dropout rates of 9% (rheumatology [35]) and 22% (sickle cell disease [36]). We understand that, in some centres patients may continue to be under the care of paediatric teams, but adult service establishment will be in a better position to manage and monitor adult medical issues [7,9].

Blood spots were returned more frequently in SP2. Some of this was associated with BH_4_ loading tests, as these involved close monitoring of blood (Phe) to accurately determine Phe tolerance variations and its impact on metabolic control [37]. However, after excluding patients enrolled for a BH_4_ loading test, there was still a tendency for increased number of blood spots, although not reaching statistical significance. In the adult care protocol, clinic visits often included the collection of blood samples by a nurse for blood Phe measurement. Thereby, it is possible that the more regular follow-up in adult care may have improved blood spot return since the frequency of appointments ranged from every 2 months to every 4–6 months. Patients were also encouraged to track their blood Phe levels at home frequently, allowing physicians and patients to better understand their biochemical phenotype. The three adult physicians were supportive of diet for life treatment, so they were fully committed to scheduling regular clinic visits and prescribing the blood Phe measurements requests, according to what the team agreed for each individual patient. Every month, the metabolic team met to discuss each patient scheduled to attend, while also sharing good practices and training between the paediatric and adult team.

There was a lower percentage of Phe measurements below 480 µmol/L in SP2. In patients who did not have a BH_4_ loading test, there was also a small increase in their median blood Phe. Statistically, this represents a slight but significant deterioration of metabolic control, although it is uncertain whether it could have significant clinical impact. We expected some deterioration of blood Phe control with increasing age [17,18,20,38,39] regardless of changes in healthcare, but moving to an adult setting may have contributed to this tendency [40]. In contrast, one short term study documented improved median blood Phe post-transfer [30], but the authors based this result on limited blood Phe measurements.

Protein intake when patients entered adult care versus after SP2 remained unchanged. There were no significant differences for daily natural protein, protein equivalent from protein substitute, total protein and Phe intakes. This suggests that patients maintained stable dietary intakes and continued to take the prescribed protein substitutes after receiving treatment in an adult setting for two years. This was not surprising as patients were seen by the same nutritionists and received consistent dietary advice. Also, the new adult team members were completely aligned with the paediatric approach to ensure the same treatment ethos was maintained in adulthood. 

In both SPs, frequency of outpatient visits exceeded the minimum requirement of two or one per year (for ages 12–18 years or ≥18 years, respectively) as recommended by the European PKU Guidelines [2]. Despite the frequent visits, however, the percentage of patients who chose not to attend appointments was very low throughout the study. Clinic visits became more frequent in SP2 due to additional nutrition appointments (5 in SP1 vs. 7 in SP2), related to the preparation process for loading tests with BH_4_ [33]. Medical follow-up was also more frequent in SP2 as most patients did not have medical appointments in SP1. Before 2013, the medics working in collaboration with *Centro de Genética Médica* did not follow-up patients with PKU frequently unless their clinical condition required medical care. The merge with *Centro Hospitalar Universitário do Porto* allowed the adult physicians to slowly start seeing more adult patients in the following years, as well as more often than in the past. The fact that the patients in this sample saw their adult physician for the first time between 2011 and 2015 therefore clarifies why most patients did not have medical follow-up in the two years prior. This lack of medical appointments also suggests that these patients did not exhibit signs of medical issues requiring close medical surveillance in this period.

We are uncertain if a team of nutritionists and psychologists specializing in adult care could potentially provide better care for adult patients, as opposed to the same professionals providing lifelong care. Adults require a more patient-focused approach, unlike paediatric care which is very family-oriented [41]. Specialists in adult healthcare may also have more training and experience dealing with specific issues of this population, such as pregnancy, depression, co-morbidities and health conditions associated with ageing. When asked about the challenges of receiving care in a paediatric facility, adult patients with PKU highlighted the lack of access to adult-oriented resources, while the main benefit was the familiarity and positive relationships with the paediatric team [22]. Leaving paediatric specialists and changing to new healthcare professionals can induce anxiety and be a major barrier for transfer [42,43], and establishing relationships with a completely new group of professionals can be overwhelming for both patients and families. Therefore, we aimed to limit the addition of new members to the adult team. We were able to keep the same nutritionists in the paediatric and adult teams; nutritionists have a pivotal role in the management of PKU.

This study had several limitations. Patient numbers were small, and all data was collected retrospectively. BH_4_ loading tests in SP2 led to more frequent clinic visits, and may have influenced frequency of blood testing, metabolic control, and adherence. No patient satisfaction surveys were conducted to assess patient acceptance of their new adult care service. These patients started follow-up with an adult physician before the paediatric and adult services were fully established. Their age also surpasses what is usually considered acceptable for a transition to adult care. As a result, these findings may not reflect the reality of patients who are currently undergoing transition at *Centro Hospitalar Universitário do Porto*. Finally, it would have been important to compare our results with other models of transition for patients with PKU.

## 5. Conclusions

The model adopted in our centre for implementing adult care for patients with PKU resulted in no significant loss of follow-up. There was, however, some deterioration in blood Phe control that should not be overlooked. Maintenance of most members of the metabolic team probably contributed to these results, but high mean age and exposure to a BH_4_ loading test added ambiguity to the outcome of this study. Prospective multicentre studies are needed to better understand the real impact of transition in patients with PKU. Future research should also address aspects such as low adherence to treatment in adults, and if new therapies will help motivate patients to remain follow-up in adult clinics post-transition.

## Figures and Tables

**Table 1:** Gender, disease severity, age and year of the first appointment with an adult physician, and enrolment to a BH_4_ loading test.

Characteristic		Total (*n* = 55)
**Gender, *n* (%)**	Female	30 (54.5)
	Male	25 (45.5)
**Disease severity, *n* (%)**	Hyperphenylalaninemia	5 (9.1)
	Mild PKU	26 (47.3)
	Classical PKU	24 (43.6)
**Age at the first appointment with an adult physician, years (mean ± SD (range))**		23.3 ± 4.3 (18–33)
**Year of the first appointment with an adult physician, *n* (%)**	2011	1 (1.8)
	2012	9 (16.4)
	2013	31 (56.4)
	2014	11 (20.0)
	2015	3 (5.5)
**BH_4_ loading test, *n* (%)**	SP1	0 (0)
	SP2	24 (43.6)

PKU, phenylketonuria; BH_4_, tetrahydrobiopterin; SP1, study period 1; SP2, study period 2.

**Table 2:** Metabolic control of patients from the final sample (*n* = 52). Results are expressed as median (P_25_–P_75_) or mean ± SD. *p* < 0.05 was considered significant.

	SP1 (*n* = 52)	SP2 (*n* = 52)	*p*
Annual number of blood spots, *n*	11 (7–15)	14 (7–20)	0.002
Median blood (Phe), µmol/L	525 ± 248	552 ± 225	0.100
(Phe) measurements < 480 µmol/L, %	51 (4–96)	37 (5–85)	0.041

SP1, study period 1; SP2, study period 2; Phe, phenylalanine.

**Table 3:** Frequency of clinic visits in SP1 and SP2 (*n* = 52). Results are expressed as median (P_25_–P_75_). *p* < 0.05 was considered significant.

	SP1 (*n* = 52)	SP2 (*n* = 52)	*p*
**Attended appointments, *n***			
Nutrition	5 (3–6)	7 (5–8)	<0.001
Medical	0 (0–1)	4 (3–5)	<0.001
Total	5 (4–6)	11 (8–13)	<0.001
**Missed appointments, *n***			
Nutrition	0 (0–1)	0 (0–1)	0.014
Medical	0 (0–0)	0 (0–1)	0.001
Total	0 (0–1)	1 (0–2)	<0.001

SP1, study period 1; SP2, study period 2.

**Table 4:** Daily protein and Phe intakes (*n* = 52). Results are expressed as median (P_25_–P_75_). *p* < 0.05 was considered significant.

	Entering Adult Care (*n* = 52)	After SP2 (*n* = 52)	*p*
Natural protein, g/kg/day	0.46 (0.35–0.88)	0.46 (0.28–0.94)	0.873
Protein equivalent, g/kg/day	0.85 (0.47–1.10)	0.83 (0.43–1.05)	0.066
Total protein, g/kg/day	1.51 (1.26–1.66)	1.34 (1.07–1.54)	0.194
Phe, mg/day	1210 (830–2311)	1318 (763–2935)	0.278

SP2, study period 2; Phe, phenylalanine.

## Data Availability

The data presented in this study are available on request from the corresponding author. The data are not publicly available due to privacy and ethical reasons.
